# Association Between Regulatory Emotional Self-Efficacy and Immunosuppressive Medication Adherence in Renal Transplant Recipients:Does Medication Belief Act as a Mediator?

**DOI:** 10.3389/fphar.2021.559368

**Published:** 2021-03-08

**Authors:** Jia Liu, Xiao Zhu, Jin Yan, Lina Gong, Xiaoxia Wu, Min Liu, Ping Mao

**Affiliations:** ^1^ Nursing Department, Third Xiangya Hospital, Central South University, Changsha, China; ^2^ Xiangya School of Nursing, Central South University, Changsha, China; ^3^ Research Center of Chinese Health Ministry on Transplantation Medicine Engineering and Technology, Changsha, China

**Keywords:** immunosuppressive medication adherence, renal transplant recipients, mediator, medication belief, regulatory emotional self-efficacy

## Abstract

**Background:** Few studies have investigated the association between regulatory emotional self-efficacy (RESE) and immunosuppressive medication adherence or the mechanisms underlying this relationship. Considering that previous evidence of immunosuppressive medication adherence depended on the level of immunosuppressive medication beliefs, a model of multiple mediation was tested in which immunosuppressive medication beliefs acted as mediators of the relationship between RESE and immunosuppressive medication adherence.

**Methods:** A retrospective cross-sectional study was performed in 293 renal transplant patients during outpatient follow-ups from November 2019 to February 2020 in China. All participants completed a general demographic questionnaire, the Chinese version of the RESE, the Beliefs about Medication Questionnaire, and the Basel Assessment of Adherence with Immunosuppressive Medication Scale (BAASIS). Spearson correlation analysis was carried out to identify the correlation between RESE and immunosuppressive medication adherence. Binary logistic regression analysis was performed to confirm factors associated with immunosuppressive medication adherence in renal transplant recipients. Mediating effect analysis was used to explore the internal interaction between RESE and immunosuppressive medication adherence.

**Results:** A total of 293 renal transplant patients were recruited, including 111 women and 182 men with a mean age of 42.5 years (SD = 10.0). A total of 23.21% of patients exhibited immunosuppressive medication none-adherence behavior, and 12.97% reported altering the prescribed amount of immunosuppressive medication without physician permission, which was most popular behavior among patients. The mean RESE score was 45.78 ± 6.12; the positive (POS) score was the highest, and the anger-irritation (ANG) score was the lowest. The correlation analysis results showed that RESE (*r* = −0.642, *p* < 0.01) and immunosuppressive medication beliefs (*r* = −0.534, *p <* 0.01) were significantly associated with immunosuppressive medication adherence. Binary logistic regression analysis indicated that marital status, fertility status, rejection, immunosuppressive medication beliefs, and RESE were found to be independent predictors of immunosuppressive medication adherence [*R* ^2^ = 0.803, *p* < 0.05]. The results of the mediating effect analysis showed that immunosuppressive medication necessity had a partial mediating effect, RESE directly and indirectly affected immunosuppressive medication adherence via immunosuppressive medication necessity, and immunosuppressive medication concerns were not a mediator between RESE and immunosuppressive medication adherence.

**Conclusion:** The levels of immunosuppressive medication adherence in renal transplant patients need to be improved in China. Marital status, fertility status, rejection, immunosuppressive medication beliefs, and RESE were major factors affecting immunosuppressive medication adherence. RESE could affect immunosuppressive medication adherence indirectly through immunosuppressive medication necessity.

## Introduction

Renal transplantation is the most effective treatment for end-stage renal disease. It is well known that renal transplant recipients need to take immunosuppressants to prevent immune rejection for the rest of their lives after surgery. It is a large challenge for them to strictly adhere to the schedule and dosage of immunosuppressive medication. Among solid organ transplant patients, renal transplant patients had the highest immunosuppressive medication non-adherence (Mellon et al., 2017), ranging from 20 to 70% (Weng et al., 2013; Liu et al., 2015; Reese et al., 2017; Paterson et al., 2018; Xia et al., 2019). Many studies have reported non-adherence (i.e., not taking medication as prescribed) as a primary reason for renal transplant failure (Weng et al., 2013; Gaynor et al., 2014; Prihodova et al., 2014). Compared with other factors, potentially modifiable factors such as social support, dialysis experience, unpleasant side effects, treatment options, attitudes towards medicine taking, forgetfulness, fatigue, self-efficacy for self-management and mental health issues played the greater roles in the immunosuppressive medication adherence of renal transplant patients (Williams et al., 2014; Jamieson et al., 2016; Nerini et al., 2016; Rebafka, 2016; Scheel et al., 2018). This fact has attracted attention among scholars.

RESE refers to the degree of confidence that an individual can effectively regulate his own emotional state, which mainly includes perceived self-efficacy in managing anger/irritation (ANG),despondency/distress (DES),and positive affect (POS)(Bandura et al., 2003; Caprara et al., 2008). Relevant studies have proved that RESE was related to aggressive behavior, violent behavior, job-hunting behavior, etc. RESE played an important role in coping with pressure, changing the interpersonal relations and bad behaviors(Annesi and Vaughn, 2017; Valois et al., 2017; Hasking et al., 2018; Mesurado et al., 2018). A few studies have shown that RESE can directly or indirectly affect a patient's behavior choices (Luque et al., 2017; Paterson et al., 2018; Yao, 2018). Patients with renal transplantation always face realistic problems that are stressful and are prone to negative emotions due to various aspects, such as heavy economic burden, fear of recurrence, unstable income, fatigue, and sleep disorders (Liaveri et al., 2017; van Sandwijk et al., 2019). However, the relationship between regulatory emotional self-efficacy and immunosuppressive medication adherence in renal transplant patients remains unclear.

Medication belief is a modifiable cognitive factor that predicts medication adherence more than clinical and sociodemographic factors (Horne, 2006; Parekh et al., 2011). Many studies have indicated that medication belief affects medication adherence, and this conclusion also applies to patients after renal transplantation, which was confirmed in our previous study (Xia et al., 2019). The Necessity-Concerns Framework (NCF) assumes that the individual’s medication adherence behavior is jointly affected by medication beliefs (including the necessity of prescribed medication and medication-related concerns) and other factors (demographic sociology, disease, psychology, society, etc.). Moreover, other relevant factors can directly or indirectly influence medication adherence behavior through medication belief (Qiao, 2018). Therefore, it is worth exploring how RESE affects medication adherence in patients after renal transplantation.

In view of this, this research proposed the following research hypothesis: 1) The RESE of renal transplant recipients was correlated with immunosuppressive medication adherence. 2) immunosuppressive medication beliefs, including immunosuppressive medication necessity and immunosuppressive medication concerns, could act as mediators to regulate the correlation between RESE and immunosuppressive medication adherence among renal transplant patients during outpatient follow-ups.

## Materials and Methods

### Design and Setting

A cross-sectional survey was conducted at the follow-up clinic of the Organ Transplantation Center of the Third Xiangya Hospital of Central South University, Changsha, Hunan Province. This study passed the ethical review of the Human Subjects Institutional Review Board at the Third Xiangya Hospital of Central South University in March 2019 (No: 2019-S161).

### Participants

A total of 293 renal transplant recipients were recruited from the follow-up outpatient clinic between October 2019 and February 2020. The inclusion criteria were as follows: 1) age ≥18 years’s old, 2) functioning renal transplant (not on dialysis), 3)Transplantation physician’s and nephrologist’s assents that recipient is able to participate in the study, 4) could speak and read Mandarin, and 5) signed an informed consent form for voluntary participation in the study. The exclusion criteria were as follows: diagnosed with severe mental illness or cognitive impairment.

## Methodological Details and Procedures

Renal transplant recipients who were eligible were invited to participate in the study and provided with information on the study objectives, study content, and investigation procedures as well as the principle of anonymity used in this study. Upon agreeing to participate in the study, informed consent papers were signed. Questionnaires were then given and completed by the participants (it took approximately 15 min to complete the questionnaires) while they waited for their clinic consultation. All investigators were trained and passed the examination. Renal transplant recipients who visited the outpatient follow-up clinic during the investigation period were recruited and completed all questionnaires in different rooms. After the questionnaires were completed, they were collected immediately and checked for missing information. Incomplete or incorrect questionnaires would be rejected.

This is a research related to medication adherence. We used BAASIS scale to assess the immunosuppressive medication adherence of renal transplant patients over the past 4 weeks. To ensure the standardization of the research process, we referred to the ESPACOMP Medication Adherence Reporting Guideline (EMERGE) and ABC taxonomy of medication adherence (De Geest et al., 2018). The initial time of taking medication was the first dose taken as prescribed 28 days ago, and the ending time was the last dose taken as prescribed on the day before the investigation. The researchers did not provide guidance or intervention for patients during 28 days.

### Measurements

A total of four questionnaires were used in this study.

## General Demographic Questionnaire

This was a self-developed questionnaire used to obtain information on general demographic variables, such as sex, age, level of education, marital status, fertility status, occupational status, length of post-transplant period, blood type, waiting time for organ donation, type of dialysis, hormone use, rejection, kind of immunosuppressive medication prescribed and so on.

## Chinese Version of the Regulatory Emotional Self-Efficacy Scale

This scale was first developed by Caprara and then adapted by Chinese scholars in 2009 according to Chinese cultural characteristics. The scale has good reliability and validity, the total Cronbach’s *α* coefficient was 0.85, and the three-dimensional Cronbach’s *α* coefficient ranged from 0.77 to 0.85. For a total of 12 items, the Likert five-level scoring method was used to set the item options from “very inconsistent” to “very consistent,” and the higher the score was, the stronger the self-efficacy of emotional regulation (Caprara et al., 2008; Wen et al., 2009).

## Beliefs About Medication Questionnaire

The Beliefs about Medications Questionnaire (BMQ) was used to evaluate the immunosuppressive medication beliefs of renal transplant patients. It was developed by Horne to assess beliefs about medicine among patients with chronic diseases, such as the qualitative interview summary of belief, which has been widely used abroad. The scale consists of two five-item scales, a total of 10 items, to assess patients’ beliefs about the necessity of prescribed medication and their medication-related concerns. All items are scored on a 5-point Likert scale from “very inconsistent” to “very consistent,” and medication belief is calculated as the difference between the necessity and concern scales, with a range of −20 to +20. A positive score indicates that the patients rated their beliefs in the necessity of taking medications higher than their concerns about the medication and vice versa (Horne and Weinman, 1999). The scale was translated into Chinese in 2014 and was previously used to evaluate medication beliefs among elderly patients with depressive disorder (Lv et al., 2014).

## Chinese Version of the Basel Assessment of Adherence With Immunosuppressive Medication Scale

The BAASIS is a self-reported questionnaire developed by the Leuven-Basel Adherence Research Group (Dobbels et al., 2010b). It examines two dimensions of medication adherence: implementation and discontinuation. Implementation was assessed by four questions (dose missing, dose skipping, timing deviation more than 2 h from prescribed time, and dose altering). A Likert six-level scoring method was used to set the item options from “never” to “more than four times”; discontinuation was assessed by one question (completely stopping medication intake). Overall, non-adherence is defined as a “Yes” answer to any of the five questions regarding implementation or discontinuation in the last 4 weeks. The BAASIS scale was translated into Chinese in 2016 and was previously used to evaluate immunosuppressive medication non-adherence among Chinese transplant recipients. The BAASIS score has also demonstrated favorable reliability; the total Cronbach’s *α* coefficient was 0.697, and the retest reliability was 0.964 (De Bleser et al., 2011; Shang et al., 2017; Shemesh et al., 2017).

### Data Analysis

All questionnaire information was entered into a computer. The data analysis was performed using SPSS 20.0 (SPSS, Inc., Chicago, Illinois, United States). Descriptive statistics are expressed in terms of frequency, percentage, mean, and standard deviation. Binary logistic regression analysis was used to find the factors affecting immunosuppressive medication adherence after renal transplantation. Spearson correlation analysis was performed to explore the correlations between RESE and immunosuppressive medication adherence.

**FIGURE 1:**
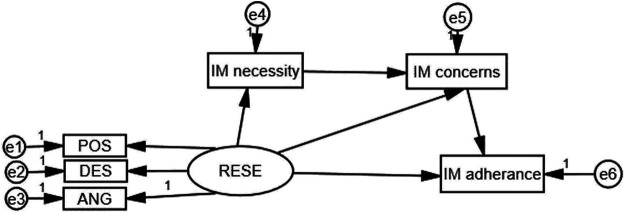
Structural model of immunosuppressive medication (IM) beliefs as a mediator of RESE and immunosuppressive medication adherence.

Amos 21.0 (Analysis of Moment Structures, IBM, Armonk, New York, United States) was used to analyze the indirect effect of immunosuppressive medication beliefs on RESE and immunosuppressive medication adherence. A structural equation model was used to examine the mediating effect of immunosuppressive medication beliefs (immunosuppressive medication concerns and immunosuppressive medication necessity) on the association between RESE and immunosuppressive medication adherence. The bias-corrected percentile bootstrap CI method was used to calculate the 95% confidence intervals (95% CIs) of the coefficients for the total, direct, and indirect effects. Coefficients were considered to be statistically significant if the 95% CIs did not cross zero (Preacher and Hayes, 2004). Statistical significance was set at *p* < 0.05; all the tests were two-sided. In our research, RESE was considered the independent variable, immunosuppressive medication adherence was considered the dependent variable, immunosuppressive medication beliefs were considered the mediating variable to construct the mediating effect model. The theoretical model was shown in Figure 1.

## Results

### Patient Characteristics

In this study, a total of 302 questionnaires were distributed, of which 293 were completed, yielding a response rate of 97.02%. More than half of the sampled patients (62.1%) were males, and the mean age of the respondents was 42.5 years (SD = 10.0 years); 32.8% had graduated from high school, and 59% were employed. Most (82.9%) were married, and 81.6% had children (Table 1).

**TABLE 1:** Patient characteristics and disease-related information (*n* = 293).

Items	*N*	%	Items	*N*	%
Sex	—	—	Dialysis before transplantation	—	—
Male	182	62.1	Hemodialysis (HD)	223	76.1
Female	111	37.9	Peritoneal dialysis (PD)	60	20.5
Age (y)	—	—	HD combined with PD	10	3.4
18–44	162	55.3	Waiting time of organ donation (y)	—	—
45–59	119	40.6	< 1	125	42.7
≧ 60	12	4.1	1 – 3	86	29.4
Education level	—	—	3 – 5	63	21.5
Primary and below	20	6.8	> 5	19	6.5
Junior middle school	74	25.3	The length of transplantation (m)	—	—
High school	96	32.8	< 6	48	16.4
Junior college	52	17.7	6 – 12	57	19.5
College degree and above	51	17.4	12 – 36	118	40.3
Occupational status	—	—	> 36	70	23.9
Employed	173	59.0	Kind of immunosuppressive medication	—	—
Unemployed	120	41.0	1	60	20.5
Marital status	—	—	2	233	79.5
Married	243	82.9	Taking hormones	—	—
Unmarried/divorced/widowed	50	17.1	Yes	150	51.2
Fertility status	—	—	No	143	48.8
Have children	239	81.6	Rejection	—	—
No children	54	18.4	Yes	53	18.1
Primary kidney disease diagnosis	—	—	No	240	81.9
Chronic nephritis	226	77.1	Hypertension	—	—
Diabetic nephropathy	13	4.4	Yes	191	65.2
Others	54	18.4	No	102	34.8

### Disease-Related Information

A total of 77.1% of patients had renal transplantation due to kidney failure caused by chronic kidney disease, and 76.1% had undergone hemodialysis. Of the participants, 42.7% received organ donation within one year, and only 6.5% waited for more than 5 years. A total of 40.3% of patients had a renal transplantation length between 1 and 3 years. A total of 65.2% had been diagnosed with hypertension before transplantation, and 18.1% had rejection; 79.5% were taking two kinds of immunosuppressive medication, and 51.2% were taking hormones (Table 1).

### Responses to the Basel Assessment of Adherence with Immunosuppressive Medication Scale for Renal Transplant Recipients

In our research, 68 (23.21%) renal transplant recipients showed non-adherence during outpatient follow-up in the last 4 weeks. Among the immunosuppressive medication non-adherence patients, 38 (12.97%)patients changed the prescribed amount; the number of patients who taking the medicine more than 2 h before or after the prescribed dosing time, missing one or more doses and skipping two or more doses were 36 (12.29%), 10 (3.41%), and 24 (8.19%), respectively. No recipients stopped taking immunosuppressive medication completely without physician permission.

### Immunosuppressive Medication Beliefs and Regulatory Emotional Self-Efficacy Outcomes

The mean score on the immunosuppressive medication beliefs scale was 16.25 ± 4.04, with a range between −2 and 20. The score for the necessity of prescribed immunosuppressive medication (22.40 ± 2.65) was higher than that for renal transplant patients’ medication-related concerns (6.15 ± 1.89). The item with the highest score was “My life would be impossible without my medicines,” and the item with the lowest score was “My medicines disrupt my life.” The total score on the RESE scale was 45.78 ± 6.12, ranging from 26 to 56. Among the three dimensions, the POS score was the highest, and the ANG score was the lowest (Table 2).

**TABLE 2:** Overall evaluation of immunosuppressive medication beliefs and RESE.

Items	Minimum	Maximum	Mean	SD
Immunosuppressive medication beliefs	−2	20	16.25	4.04
Immunosuppressive medication necessity	11	25	22.40	2.65
Immunosuppressive medication concerns	5	20	6.15	1.89
RESE	26	56	45.78	6.12
POS	5	20	15.65	2.37
DES	8	19	15.38	2.62
ANG	7	19	14.76	2.38

### Correlations Among Immunosuppressive Medication Adherence, Beliefs and Regulatory Emotional Self-Efficacy

There were significant correlations among immunosuppressive medication adherence, immunosuppressive medication beliefs, and RESE. The immunosuppressive medication adherence score was negatively correlated with the RESE score, POS, DES and ANG (*r* = −0.642 to −0.556, *p* < 0.01). The immunosuppressive medication beliefs score was negatively associated with immunosuppressive medication adherence (*r* = −0.534, *p* < 0.01) and positively associated with the RESE score (*r* = 0.449, *p* < 0.01). The relationships were all in the expected direction: better immunosuppressive medication adherence was associated with better RESE and immunosuppressive medication beliefs. In addition, the patients with lower levels of immunosuppressive medication necessity and higher levels of immunosuppressive medication concerns indicated worse immunosuppressive medication adherence (Table 3).

**TABLE 3:** Correlations among immunosuppressive medication adherence, beliefs and RESE.

Items	Immunosuppressive medication adherence	Immunosuppressive medication beliefs	Immunosuppressive medication necessity	Immunosuppressive medication concerns	RESE	POS	DES	ANG
immunosuppressive medication adherence	1							
immunosuppressive medication beliefs	−0.534**	1						
immunosuppressive medication necessity	−0.526**	0.922**	1					
immunosuppressive medication concerns	0.478**	−0.841**	−0.567**	1				
RESE	−0.642**	0.449**	0.467**	−0.305**	1			
POS	−0.589**	0.332**	0.337**	−0.236**	0.846**	1		
DES	−0.556**	0.405**	0.422**	−0.274**	0.844**	0.589**	1	
ANG	−0.603**	0.380**	0.401**	−0.248**	0.801**	0.532**	0.483**	1

**p < 0.01.

### Factors Predicting Immunosuppressive Medication Adherence

Before binary logistic regression analysis, we examined the relationship between patient characteristics and immunosuppressive medication adherence through univariate analysis,to screen out some variables that were meaningless. Finally, variables of patient characteristics—namely, age, marital status, fertility status, dialysis before transplantation, rejection and RESE, immunosuppressive medication beliefs, were included in the binary logistic regression analysis as independent variables. Table 4 shows the independent variables assignment of binary logistic regression analysis of renal transplant patients’ immunosuppressive medication adherence. Table 5 shows the results of the binary logistic regression analysis identifying signifcant factors that predict medication adherence. The model can explain 80.3% of the change in the medication adherence level. Marital status, fertility status, rejection, RESE, immunosuppressive medication beliefs could predict renal transplant recipients medication adherence significantly (*p < 0.05*). It indicated the patients who were married, had no children, without rejection, and had higher levels of immunosuppressive medication beliefs and RESE were more likely to be adherent to immunosuppressive medication.

**TABLE 4:** Independent variables assignment of binary logistic regression analysis of renal transplant patients’ immunosuppressive medication adherence.

Independent variables	Assignment
Age	18 – 44 = 1; 45–59 = 2; ≧ 60 = 3
Marital status	Married = 1; Unmarried/divorced/widowed = 2
Fertility status	Have children = 1; No children = 2
Dialysis before transplantation	Hemodialysis (HD) = 1; Peritoneal dialysis (PD) = 2; HD combined with PD = 3
Rejection	Yes = 1; No = 2
RESE	Continuous value
Immunosuppressive medication beliefs	Continuous value

**TABLE5:** Binary logistic regression analysis for factors predicting immunosuppressive medication adherence.

Variables	B	SE	Odds ratio	95%CI	*p*-value
Marital status	3.669	1.129	39.224	4.294–358.335	0.001
Fertility status	−2.291	1.088	0.101	0.012–0.854	0.035
Rejection	−1.667	0.699	0.189	0.048–0.743	0.017
RESE	−0.405	0.060	0.667	0.593–0.750	0.000
Immunosuppressive medication beliefs	−0.294	0.065	0.745	0.656–0.845	0.000

### Medication Beliefs Acting as Mediators

**FIGURE 2:**
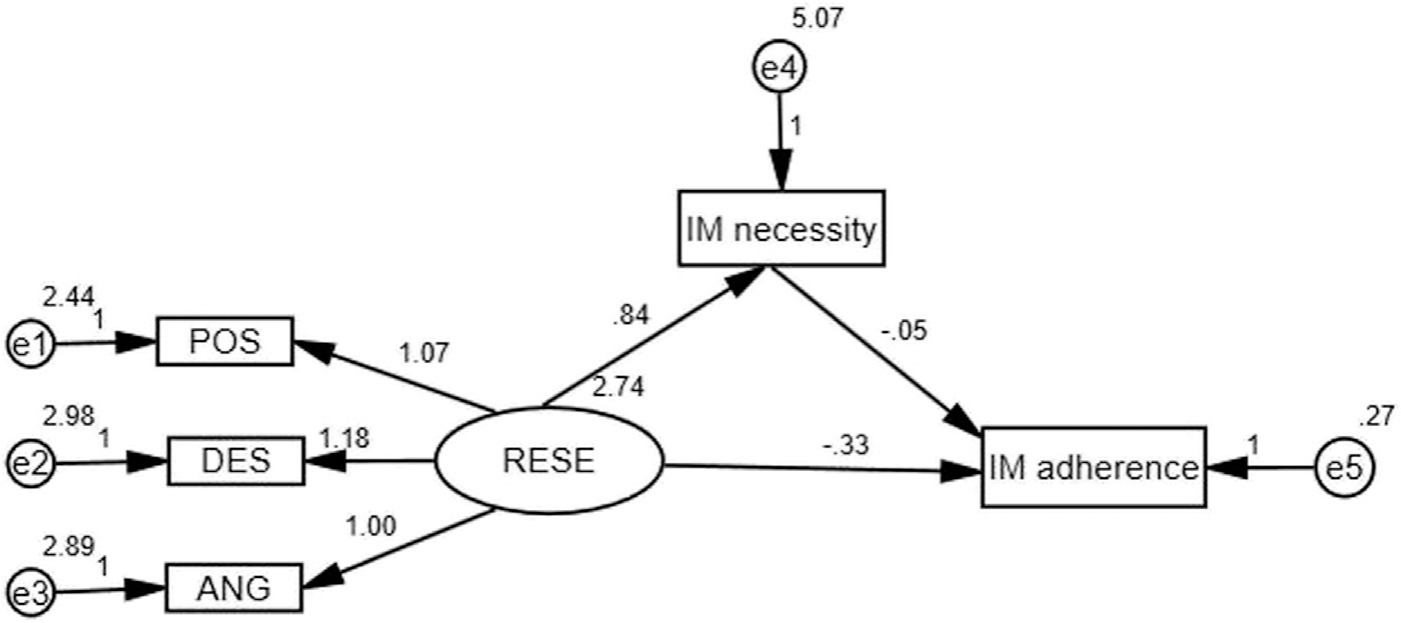
The mediation model of immunosuppressive medication (IM) necessity.

The initial model was a good fit to the data, with χ^2^/df = 1.541, GFI = 0.990, CFI = 0.995, and RMSEA = 0.043, *p* = 0.160. The modeling results indicated that there was no significant effect among the paths, including the following paths: RESE→immunosuppressive medication concerns, immunosuppressive medication concerns→immunosuppressive medication adherence (*p* > 0.05). This also implied that immunosuppressive medication concerns were not a mediated variable. Therefore, we modified the model and deleted the above three paths, as shown in Figure 2. The modified model had goodness-of-fit indices as follows: χ^2^(4) = 9.032, *p* = 0.060 > 0.05, χ^2^/df = 2.258, GFI = 0.988, CFI = 0.990, and RMSEA = 0.066. The results of the bootstrap analyses further indicated that RESE was significantly associated with immunosuppressive medication necessity (direct effect *β* = 0.838, 95% CI = 0.609∼1.072, *p* < 0.001) and that immunosuppressive medication necessity was significantly associated with immunosuppressive medication adherence (direct effect *β* = −0.055, 95% CI = −0.107 to −0.008, *p* = 0.001). RESE was significantly associated with immunosuppressive medication adherence (total effect: *β* = −0.380, 95% CI = −0.465 to −0.296, *p* < 0.001; direct effect *β* = −0.334, 95% CI = −0.431 to −0.240, *p* < 0.001), and immunosuppressive medication necessity could act as a mediator to regulate the relation between RESE and immunosuppressive medication adherence (*β* = −0.046, 95% CI = −0.093 to −0.009, not including “0”).

## Discussion

Taking immunosuppressive medication as prescribed for life is critical to maintaining transplant kidney function (Griva et al., 2018). Strengthening the drug management of renal transplant patients and improving their medication adherence are still worthy of the focus of the present research. The goal of the present study was to examine the relationship between RESE and immunosuppressive medication adherence in a sample of renal transplant patients and to explore whether immunosuppressive medication beliefs act as a mediator in that relationship at the same time. In our study, we found that RESE was strongly correlated with immunosuppressive medication adherence and that RESE could indirectly affect immunosuppressive medication adherence through immunosuppressive medication necessity.

Numerous studies have indicated that the incidence of immunosuppressive medication non-adherence in renal transplant patients ranges from 20 to 70% (Weng et al., 2013; Liu et al., 2015; Reese et al., 2017; Paterson et al., 2018; Xia et al., 2019). This may be due to the difference in immunosuppressive medication adherence measurement tools and assessment criteria in renal transplant patients. In this study, 23.21% (68) patients reported immunosuppressive medication non-adherence. The data were in the below of the reported data range. All the participants of our study came from the same center. This center has been committed to the research on immunosuppressive medication adherence of patients after transplantation for many years, and continues to provide related health education services for patients. So, the long-term health education may contribute to immunosuppressive medication adherence of transplant patients. Among the immunosuppressive medication non-adherence behaviors, the most frequent was changing the prescribed amount without physician guidance (12.97%), followed by taking a dose more than 2 h before or after the prescribed time (12.29%). This result was different from others. In many studies, including our group’s previous research, the most common non-adherence behavior was taking a dose more than 2 h before or after the prescribed time (Cossart et al., 2019; Xia et al., 2019). In our study, among the 38 patients who had changed the prescribed amount of immunosuppressive medication without doctor's permission, 32(84.21%) were more than half a year after surgery. In this period, the patients were in a stable state of illness, felt generally well, and may choose to reduce the dose of the immunosuppressive medication in consideration of the side effects of the immunosuppressive medication. Besides that, 32 (84.2%) received organ donation within one year. The short waiting time for the donor may result in the patients not cherishing the donor, insufficient understanding of the importance of prevention of rejection, and poor medication adherence. We all know that taking immunosuppressive medication as prescribed was important for maintaining appropriate immunosuppressive medication blood concentrations and preventing rejection. It was an indirect reminder to us to pay more attention to the problem of patients adjusting the dosage without physician permission and to incorporate this into the focus of follow-ups in the future.

For immunosuppressive medication beliefs, the score of immunosuppressive medication necessity was higher than the score of immunosuppressive medication concerns. This indicated that the perceived necessity outweighs the concerns among renal transplant patients. This was confirmed in a meta-analysis conducted in Germany (Bunemann et al., 2020). In addition, correlation analysis showed that the immunosuppressive medication beliefs score was negatively associated with immunosuppressive medication adherence, the immunosuppressive medication necessity score had a positive correlation with immunosuppressive medication adherence, and immunosuppressive medication concerns had a negative correlation with immunosuppressive medication adherence. When patients have greater immunosuppressive medication necessity and fewer immunosuppressive medication concerns, they may have better immunosuppressive medication adherence, which is similar to trends in other diseases (Foot et al., 2016).

The risk factors associated with immunosuppressive medication non-adherence in adult and pediatric kidney transplant patients could classify into five categories broadly: socioeconomic factors, condition-related factors, patient-related factors, treatment-related factors, and factors related to the healthcare system (Dobbels et al., 2010a; Belaiche et al., 2017). In our study, marital status, fertility status, rejection, immunosuppressive medication beliefs and RESE significantly predicted immunosuppressive medication adherence. Patients who were married and had better immunosuppressive medication beliefs showed better immunosuppressive medication adherence, consistent with our previous findings. However, the two studies used different assessment tools for immunosuppressive medication beliefs: the former used a self-developed immunosuppressive medication beliefs questionnaire, while the present study used the Chinese version of the BMQ (Xia et al., 2019). Compared to unmarried recipients, married recipients were more likely to adhere to medication after transplantation. This may be related to marital status being associated with positive clinical outcomes (Ladin et al., 2018). Fertility status was rarely mentioned in studies on medication adherence in transplant patients. However, our research investigated patients’ fertility status and the result showed that fertility status predicted immunosuppressive medication adherence. Patients with children had worse immunosuppressive medication adherence. This may be caused by caring for the children and forgetting to take medication. So, we could consider the fertility status as a variable in future studies on medication adherence of transplant patients and conduct further studies.

Sexual status, length of transplantation, education level, and occupational status had no significant impact on immunosuppressive medication adherence, which conflicts with the results of other studies. The results of a systematic review of 37 studies revealed that male sex, unemployment, and low education were associated with non-adherence (Belaiche et al., 2017). Regarding the length of transplantation, some studies have examined the correlation between the length of transplantation and immunosuppressive medication adherence but with different results. Patzer et al. reported that fewer months since transplantation were associated with non-adherence (Patzer et al., 2016). However, Lee et al. found that a longer time since renal transplantation was associated with low medication adherence (Lee et al., 2015).

The results also showed that patients who experienced transplant rejection had worse immunosuppressive medication adherence. It is well known that tacrolimus and cyclosporine are commonly used to prevent the occurrence of rejection. Patients are required to take drugs regularly and quantitatively as prescribed by physicians. Many studies have found that immunosuppressive medication non-adherence can lead to an increased risk of rejection (Belaiche et al., 2017; Leven et al., 2017). Of course, the occurrence of rejection is not just related to immunosuppressive medication non-adherence, and longer pre-transplant dialysis duration, HLA mismatch, and positive pre-transplant PRA were also risk factors for acute rejection (Fu et al., 2018).

Among the three dimensions of RESE, ANG score was the lowest in our 293 renal transplant patients, indicated that the recipients had a poor ability to regulate anger/anger emotion. In addition, the RESE, POS, DES, and ANG scores were significantly negatively correlated with the immunosuppressive medication adherence score. This implied that the stronger ability of RESE, the better the immunosuppressive medication adherence was, which was similar to results reported by several studies (Luque et al., 2017; Wang et al., 2017). RESE was correlated with immunosuppressive medication beliefs, and immunosuppressive medication beliefs were also correlated with immunosuppressive medication adherence based on previous research results. Based on this, we had more confidence in our second hypothesis that immunosuppressive medication beliefs had a mediating effect on RESE and immunosuppressive medication adherence. Our study partially confirmed this hypothesis. In our study, immunosuppressive medication beliefs included two parts: immunosuppressive medication necessity and immunosuppressive medication concerns. Since we do not know whether these two variables both had a mediating effect or one of them had a mediating effect, we built a multiple mediation effects model. By running the model, we discovered that RESE could affect immunosuppressive medication adherence directly or indirectly through immunosuppressive medication necessity, and the former played a major role. Some previous studies have demonstrated that RESE could regulate individual behaviors. Penelope et al. showed that regulatory emotional self-efficacy was a salient predictor of self-injury and disordered eating, evidencing both direct and indirect relationships (Hasking et al., 2018). The results of a study performed in Spain found that RESE had a direct relationship with prosaic behavior and aggression (Luque-Reca et al., 2016). Considering the long course of disease, many complications and heavy economic burden, renal transplant recipients were prone to have negative emotions such as anxiety, depression, irritability and irritability. This suggested that it was necessary to detect the adverse emotions of renal transplant recipients earlier evaluate the ability to cope with adverse emotions,screen out the inappropriate methods or errors in dealing with adverse emotions timely, and analyze the personalized reasons, provide positive psychological interventions and social support, and enhance their awareness of emotional regulation. Through the above methods, to help renal transplant patients adopt correct and scientific emotional regulation methods, enhance their ability and confidence in resisting bad emotions, seek psychological counseling, accept psychological intervention, improve their level of RESE, and thus improve their immunosuppressive medication adherence.

In the structural equation model, immunosuppressive medication necessity had a weak mediating effect on the association between RESE and immunosuppressive medication adherence. While paying attention to the emotional regulation of renal transplant patients, we could also guide immunosuppressive medication taking behavior with immunosuppressive medication beliefs, especially immunosuppressive medication necessity, to improve immunosuppressive medication adherence. Medical staff should identify patients’ medication problems early, strengthen patients’ health education, and use multiple media channels to transfer drug knowledge to patients so that renal transplant patients can understand the necessity and benefits of taking immunosuppressive medication according to their physicians’ orders and the adverse consequences brought by non-adherence behaviors. In addition, this approach could be used to inform patients of the correct way to cope with adverse drug reactions and reduce patients’ medication concerns and anxiety; thus, patients would use positive emotion regulation methods to improve immunosuppressive medication adherence.

Admittedly, our study still had some limitations: 1) This study was a single-center, cross-sectional survey, and all of the participants were recruited from an organ transplantation center in Changsha City, Hunan Province; 2) We had a limited number of patients for this study; 3) All measurement tools were self-reported; therefore, the results for patients with immunosuppressive medication adherence and RESE were subjective and might not be convincing; 4) Most of our participants were younger than 60 years; thus, the conclusions of our study might not be suitable for patients over 60 years old. Despite the limitations, we believe our findings are significant in providing direction to the future. In the future, multicenter studies with large samples of different age groups and some objective measures of immunosuppressive medication adherence, such as biochemistry indicators, are needed to confirm our conclusions.

## Conclusion and Implications

In summary, renal transplant patients’ immunosuppressive medication adherence still needs to be improved. RESE had a direct relationship with immunosuppressive medication adherence, and RESE could also affect immunosuppressive medication adherence indirectly through immunosuppressive medication necessity. Renal transplant patients who were married, had no children, who were not experiencing transplant rejection, and who had a high level of immunosuppressive medication necessity and RESE had better immunosuppressive medication adherence than other renal transplant patients.

To improve the immunosuppressive medication adherence of patients after renal transplantation, health-care professionals and caregivers should identify patients’ emotional changes timely, especially negative emotions, provide enough family and social support, and supply psychological interventions to enhance confidence in taking medication. Moreover, health education should be strengthened, especially to improve patients’ awareness of the necessity of taking immunosuppressive medication, emphasize subjective initiative, and promote self-health management among renal transplant patients. Future studies could focus on taking effective interventions to improve the ability of emotions regulation and the awareness of immunosuppressive medication necessity, so as to improve medication adherence of transplant patients.

## Data Availability

The raw data supporting the conclusions of this article will be made available by the authors, without undue reservation, to any qualified researcher.
